# Understanding the experience of prescription charges in people living with parkinson’s disease: a focus group study

**DOI:** 10.1186/s12889-025-24121-0

**Published:** 2025-08-22

**Authors:** Megan Rose Readman, Ayomide Oluseye, Lisa Jane Brighton, Megan Polden, Ian Fairman, Ian Parkinson, Caroline Parkinson, Clarissa Giebel

**Affiliations:** 1 Department of Primary Care and Mental Health, The University of Liverpool, Liverpool, UK; 2 NIHR Applied Research Collaboration North West Coast, Liverpool, UK; 3 Department of Psychology, Lancaster University, Lancaster, UK; 4 Faculty of Wellbeing, Education and Language Studies, The Open University, Milton Keynes, UK; 5 Cicely Saunders Institute of Palliative Care, Policy and Rehabilitation, King’s College London, London, UK; 6 Division of Health Research, Lancaster University, Lancaster, UK

**Keywords:** *Prescriptions*, Prescription charge policy, *Parkinson’s disease*, Public health

## Abstract

**Background:**

In England, people aged > 60 are typically required to pay for their prescriptions. Whilst exemption criteria enable people living with specified long-term health conditions to receive free prescriptions, Parkinson’s disease is omitted from this list. People with Parkinson’s are often reliant upon medications, and evidence suggests that medical fees can reduce quality of life and medicine adherence. We, therefore, aimed to explore the impact of prescription charges on people with Parkinson’s and their family care partners (caregivers).

**Methods:**

This is a qualitative focus group study with people with Parkinson’s and caregivers. Focus groups were semi-structured and conducted online. Participants were recruited through opportunity sampling. Eligible participants were adults aged 18 and over living in England who either (1) had a diagnosis of Idiopathic Parkinson’s Disease or (2) provided unpaid care for someone with Parkinson’s, including parents, adult children, siblings, or close friends. Data was analysed using reflexive thematic analysis within a critical realist paradigm.

**Results:**

Five focus groups were conducted with people with Parkinson’s (*n* = 12) and caregivers (*n* = 12). All focus groups comprised both people with Parkinson’s and caregivers. Thematic analysis identified three overarching themes: (1) The financial toll of medication and its ripple effects; (2) Lack of inclusion and support; and (3) Difficulties of seeking support. People affected by Parkinson’s disagreed with current policy and suggestions of per-prescription charge re-evaluation were expressed.

**Conclusions:**

Prescription charges have multifaceted negative impacts on people affected by Parkinson’s. Current prescription charge policies, including their exemption criteria, should be reviewed, alongside initiatives to raise awareness of existing financial support systems, such as pre-payment certificates.

**Registration:**

Study protocol and analysis strategy are pre-registered on Open Science Framework (https://osf.io/y8ve5/).

**Supplementary Information:**

The online version contains supplementary material available at 10.1186/s12889-025-24121-0.

## Background

Parkinson’s disease, hereafter Parkinson’s, is a prevalent progressive neurodegenerative disorder that primarily presents with features of tremor, rigidity, and globally slowed movement initiation [1]. In 2019 it was estimated that approximately 8.5 million people live with Parkinson’s worldwide [2]. While increasing age is one of the largest risk factors for Parkinson’s [3], up to 5% of people living with Parkinson’s are diagnosed before the age of 50 [4]. Specifically, in 2015 approximately 10, 800 people under the age of 60 were living with Parkinson’s in the UK alone [5]. Pharmacological intervention, in particular levodopa therapy, has remained the gold standard treatment for Parkinson’s since the late 1960 s [6]. Indeed, between 62 and 81% of people living with Parkinson’s are on some form of pharmacological intervention [7, 8]. Due to the heterogeneity of Parkinson’s symptoms and the side effects associated with some anti-parkinsonian medication polypharmacy is commonplace in Parkinson’s. A recent UK-based cohort study observed that 44.5% of people with Parkinson’s were prescribed, and taking, between 5 and 9 pharmacological interventions per day, and 19.2% of people with people with Parkinson’s were prescribed, and taking, more than10 different pharmacological interventions per day [9].

Presently in England, individuals are required to pay a charge of £9.65 per prescribed medication, unless they meet pre-specified charge exemption criteria [10]. Current exemption criteria include people of specific ages, people of specific financial insecurity and people who live with pre-specified long-term health conditions [11] (See Table 1 for full exemption criteria). Parkinson’s is not, currently, included in the medical exemption criteria. Subsequently, people living with Parkinson’s aged < 60 are required to pay for their prescriptions. Should the 5–10 prescribed medications per day equate to 5–10 prescriptions per month this could amass to between £46.75 and £93.5 per month.

**Table 1:** Current National Health Service (NHS) per-prescribed item charge exemption criteria applied in England

**Age based criteria**	• Under 16
	• Aged 16–18 and in full time education
	• Aged 60 or over
**Benefits based criteria**	• If you receive income Support
	• If you receive income-based Jobseeker’s Allowance
	• If you receive income-related Employment and Support Allowance
	• If you have a HC2 certificate
**Tax credit based criteria**	• If your annual family income is £15,276 or less and you receive either:
	(1) Child Tax Credit
	(2) Working Tax Credit and Child Tax Credit paid together
	(3) Working Tax Credit including a disability element
**Pregnant women and those who have had a baby in the last 12 months**	
**MedEx Criteria**	• A permanent fistula (for example, caecostomy, colostomy, laryngostomy or ileostomy) which needs continuous surgical dressing or an appliance
	• A form of hypoadrenalism (for example, Addison’s Disease) for which specific substitution therapy is essential
	• Diabetes insipidus and other forms of hypopituitarism
	• Diabetes mellitus, except where treatment is by diet alone
	• Hypoparathyroidism
	• Myasthenia gravis
	• Myxoedema (that is, hypothyroidism which needs thyroid hormone replacement)
	• Epilepsy which needs continuous anticonvulsive therapy
	• A continuing physical disability which means you cannot go out without the help of another person
	• Cancer and are undergoing treatment for either:
	(1) cancer
	(2) the effects of cancer
**People who receive War Pension Scheme or Armed Forces Compensation Scheme payments**	

To mitigate the financial burden of per-prescribed item prescription charges for people who do not meet the prescription charge exemption criteria, the Department of Health and Social Care introduced a prescription pre-payment certificate. By enabling individuals to make a one-off payment, £111.60 for 12 months or £31.25 for 3 months, that covers all per-prescribed item charges for the following year/3-month period, prescription pre-payment certificates limit the amount people pay annually for prescriptions [12]. However, prior evidence has shown that prescription pre-payment certificate awareness and use is low-moderate [13]. Moreover, although many people with long-term health conditions are aware of the prescription pre-payment certificate, they typically do not learn of the prescription pre-payment certificate for on average six months to one year following diagnosis [14].

Prior evidence suggests that financial strain can be a significant barrier to medication adherence [15]. That is, individuals may either elect not to engage with a given treatment plan, by not collecting medications or may alter their treatment plan to make it last longer, by skipping medication doses or half-dosing, due to the costs associated with the given treatment [16]. This has also been shown for people living with dementia for example, having to decide whether to pay for their social care and support services or paying instead for basic personal necessities during the recent cost of living crisis [17]. Medication non-adherence is associated with reductions in functional abilities and quality of life, the occurrence of additional health complications, premature death, and increased use of medical resources through hospitalisation [18, 19]. Thus, the financial pressures associated with per-prescribed item charges may lead people with Parkinson’s to change their medication habits, potentially resulting in them experiencing poorer health outcomes. Research also suggests that financial stress related to medical costs can lead to emotional strain and mental health issues [13, 20, 21]. For people with Parkinson’s, the reliance on prescription medications combined with these financial burdens may increase emotional distress and negatively impact their quality of life.

Although prior evidence has independently shown the reliance on medication in people with Parkinson’s [9] and the impact of the cost of medications across a range of individuals with long-term health conditions [13, 18–21], no prior literature has specifically examined the impact of per-prescribed item prescription charges on people with Parkinson’s and their family caregivers. Thus, this study aimed to explore the experiences and impacts of prescription charges for people living with Parkinson’s disorder and their caregivers.

## Methods

This study’s protocol and planned analyses can be found on the Open Science Framework (OSF; https://osf.io/y8ve5/). We summarise implementation following the Consolidated criteria for reporting qualitative studies (COREQ) criteria [22] below (see supplementary material for COREQ checklist).

Minor amendments from the pre-registered protocol included the proportions of people with Parkinson’s and caregivers recruited and variable transformations for participant characteristics. Full justifications for these deviations can be found in the supplementary materials and information regarding pre-registered variable transformations can be found in the OSF documentation (https://osf.io/y8ve5/).

### Sampling and recruitment

People with Parkinson’s were eligible to participate if they were residing in England, aged 18 and over, and had a known diagnosis of Idiopathic Parkinson’s Disease. Due to the differences in the clinical management of alternative types of Parkinson’s and Parkinsonism’s, compared to idiopathic Parkinson’s, people living with any other form of Parkinson’s including drug-induced Parkinsonism or Vascular Parkinsonism, or Parkinsonism including Multiple Systems Atrophy and Progressive Supranuclear Palsy were not eligible to participate.

Caregivers were eligible if they lived in the UK, were 18 or older, and provided unpaid care for someone with Parkinson’s. This could include parents, adult children, siblings, or other close relatives or friends.

Participants were recruited via opportunity sampling through social media advertisements, email adverts to local research networks, and via advertisement on the Parkinson’s UK national research participation hub, the Take Part Hub.

### Data collection

This study used a focus group design to explore the impact of per-prescribed item charges on people with Parkinson’s and their caregivers. Focus groups were chosen because their interactive nature can reveal insights that individual interviews might miss [23, 24], particularly when discussing sensitive topics like Parkinson’s and pre-prescribed item charges.

Focus groups were semi-structured. The focus group topic guide (see supplementary materials) was co-developed alongside the project public advisory team, including two people with Parkinson’s (IF and CP) and one family member of a person with Parkinson’s (IP). Co-development took place in three steps: (1) A draft topic guide was created based on gaps in the existing literature (MR and MP), (2) public advisers reviewed the guide and provided feedback on whether it reflected their lived experience, (3) the topic guide was revised based on the public adviser’s feedback (MR). The final topic guide asked about experiences regarding per-prescribed item charges in the context of Parkinson’s, the impact of per-prescribed item charges on themselves and their families, what the issues are surrounding per-prescribed item prescription charges in the context of Parkinson’s and what they would like to see improved.

All focus groups were conducted online, maximising the geographical spread of the participants within the UK. Focus groups lasted between 38 and 60 min in duration and were conducted between August- October 2023. All focus groups contained a mix of people living with Parkinson’s and caregivers, with the distribution of participants to focus groups being based on convenience. Focus groups were facilitated by one of two female researchers with four being facilitated by MR, and one being facilitated by MP. Both MR and MP have prior experience and training in facilitating qualitative interviews and focus groups. Each worked with the same topic guide and discussed their reflections with each other after each focus group. Only one facilitator and study participants were present for the duration of the focus group. The focus groups were audio-recorded, transcribed verbatim, and anonymised to maintain confidentiality. All focus groups were transcribed by MR and checked for accuracy by both MR and AO. To further protect the confidentiality all participants were asked to refrain from disclosing personally identifiable information, such as names, for the duration of the focus group. Prior to conducting the focus group, all participants provided written informed consent by completing an online form, informed consent was again verbally obtained at the start of the online call. Participants completed a brief demographics form, and where relevant provided further information relating to their Parkinson’s diagnosis including years since diagnosis, age at time of diagnosis, whether they take medication, the number of medications and quantity of tablets taken per day. To support data analysis both facilitators took detailed field notes describing contextual factors, participant responses, and initial reflections immediately after each focus group. No repeat interviews were conducted.

Data collection continued until the dataset was assumed to reach thematic saturation. Thematic saturation was defined as the point at which no new information, codes or themes are yielded from additional data [25]. Thematic saturation was first assessed for participant groups independently, for example, we defined saturation for data from people with Parkinson’s as the point at which no new information was generated by people with Parkinson’s. We then assessed thematic saturation across all participants, i.e. the point at which no new information was discussed from any participant regardless of group.

### Data analysis

 Data was analysed using Braun and Clarke’s reflexive thematic analysis [26]. This approach was chosen as it allows for a flexible, in-depth analysis of qualitative data, suitable for capturing participants’ diverse experiences and perspectives. We followed an inductive approach, grounded in social constructionism. By using this approach, we were able to let themes emerge naturally from the data without imposing any preconceived theories. This helped ensure that our findings remained closely grounded in the actual language and experiences of participants, which was crucial for exploring a complex and sensitive topic.

The analysis comprised five steps: (1) Data familiarisation, through reading and re-reading of transcripts, (2) simultaneous line-by-line manual coding of the transcripts independently by two researchers (AO and MR), (3) searching and grouping codes to generate overarching themes and sub-themes. Using a table, the researchers [AO and MR] were able to visually present the data and consider the links and relationships between the themes. Afterwards, (4) identified themes were then discussed with the wider team (MR, AO, LJB, IF, CP, IP and MP) to systematically refine the analysis and ensure that the themes were reflective of the data generated as a whole. During this stage, further coding and re-coding of the themes and sub-themes took place, reflecting the iterative process of data analysis. Finally, (5) themes were named, their narratives defined, and a summary table was produced with extracts from participant’s transcripts. Thematic analysis was conducted using Microsoft Excel. Table 2 shows the coding tree identifying the themes and sub-themes generated during the analysis.

**Table 2:** Coding tree: themes and associated sub-themes identified from focus groups

Theme	Sub-themes
The financial toll of medication and its ripple effects	Financial burden.
	Lifestyle sacrifices.
	Medication non-adherence.
Lack of inclusion and support	Failed by the system.
	Inconsistencies in policy.
	Limited and by-chance awareness of support.
Difficulties of seeking support	Reliance on family support. The burden of seeking support.

#### Reflexivity

Reflexivity is addressed with respect to the four facets of trustworthiness outlined by Guba et al. [27]. In addressing the credibility of our findings, while we did not engage in member checking, identified themes were actively presented to two people with Parkinson’s (IF and CP) and one caregiver (IP) for their feedback and validation. Clear contextual descriptions of the study setting, interview process and participant recruitment are provided to aid transferability. In terms of dependability, the study’s protocol, including the focus group topic guide, and planned analysis were preregistered on the OSF (https://osf.io/y8ve5/). This transparency allows for scrutiny and serves as a resource for other researchers who wish to replicate the study or build upon its findings. Regarding confirmability and preconceptions of the researchers, MRR, is a research fellow, with a background in psychology, who has worked with people living with Parkinson’s for numerous years. AO is a qualitative and creative methods researcher, and lecturer, with a background in public health, primarily interested in public health perspectives. LJB is a qualitative researcher, and lecturer, with a background in psychology, whose research focuses on people living with advanced disease, particularly respiratory conditions. MP is a research fellow, with a background in psychology, whose research primarily focuses on people living with Dementia. Considering prior relationships with participants, three people with Parkinson’s, who participated in this study, have previously participated in MRR’s research studies, the remaining of the participants were unknown to MRR and all participants were unknown to the rest of the research team. The prior studies that these participants had participated in focused on visual perceptual processes [28, 29], thus the present topic had not been previously discussed with these participants., Both facilitators kept a reflexive diary to reflect on and understand their role during the interview. During the analysis phase, MRR, AO, LJB and MP actively discussed how MRR’s experiences might influence the analysis and ensured they frequently returned to the data and sought the input of public members to strengthen interpretive rigour. Participants were naive to any personal reasons the researchers may have for conducting the research. Moreover, the facilitators ensured they remained impartial and did not discuss their personal reflections throughout the focus group.

### Patient and Public involvement

Three members of the public, public advisers, two people with Parkinson’s (IF and CP) and one caregiver (IP), were involved in the design of the research question, development of the focus group topic guide, interpretation of the analysis, and dissemination. To do so public advisers engaged in informal meetings and provided written feedback on the focus group schedule and this manuscript.

### Ethics

This study received ethical approval from the University of Liverpool Ethics Committee (reference number: 12774) and was conducted in accordance with the principles outlined in the Declaration of Helsinki. All participants provided informed consent, for their participation and for the focus group to be audio recorded and received a £25 shopping voucher reimbursement.

## Results

Thematic saturation was deemed to be reached after five focus groups comprising 24 participants (12 people with Parkinson’s and 12 caregivers). All participants that expressed interest in this study participated, that is no participants dropped out or declined participation.

**Fig. 1:**
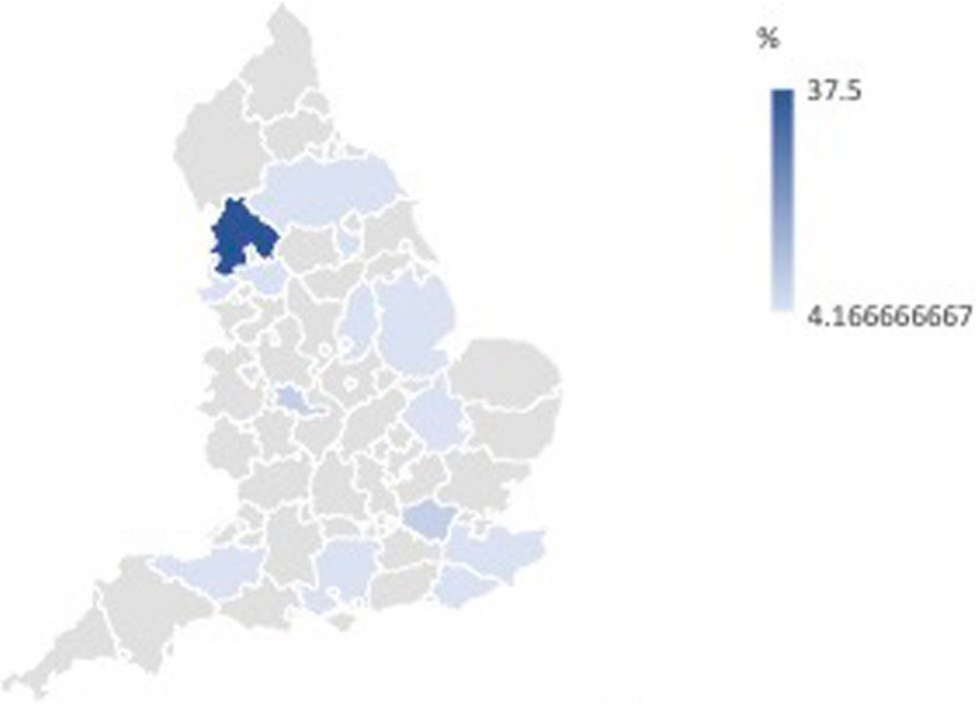
Geographical representation of participants across England

Participant demographics can be found in Table 2. However, in brief, participants were predominantly female (62.5%) from a white ethnic background (70.83%), educated up to a degree level or equivalent (54.17%), and retired (50%) or working part-time (41.67%). Participants were aged between 25 and 77 (*M* = 54.29(16.38)). Most participants were classified as average in terms of deprivation (quintile 3, 58.34%). Participants’ homes of residence spanned most of England, however, due to the use of opportunity sampling, there was a cluster of participants in the northwest coast region (See Fig. 1).

**Table 3:** Sample demographics

	People with Parkinson’s (*n* = 12)	Caregivers (*n* = 12)	Total Sample (*n* = 24)
*N*			
Age *(mean (SD))*	59.42 (7.04)	49.17 (21.32)	54.29(16.38)
Gender			
Female	6	9	15
Male	6	3	9
Ethnicity			
White	10	7	17
Black and minority ethnicities	2	5	7
Employment Status			
Working full time		2	2
Working part time	5	5	10
Retired	7	5	12
Occupation			
Managers, directors and senior officials	2 (16.67)	2 (16.67)	4
Professional occupations	3 (25)	1 (8.32)	4
Associate professional occupations	2 (16.67)		2
Administrative and secretarial occupations	1 (8.32)	2 (16.67)	3
Skilled trades occupations	2(16.67)		2
Caring, leisure and other service occupations		3 (25)	3
Sales and customer service occupations		2 (16.67)	2
Elementary occupations		1 (8.32)	1
Prefer not to say	2 (16.67)	1 (8.32)	3
IMD***			
1	2 (16.67)		2 (8.33)
2		5 (41.67)	5 (20.83)
3	7 (58.33)	7 (58.33)	14 (58.34)
4	2 (16.67)		2 (8.33)
5	1 (8.33)		1 (4.17)
Education			
Degree level or above	8 (66.67)	5 (41.67)	13 (54.17)
AS, A levels or equivalent	1 (8.33)	3 (25)	4 (16.67)
GCSEs or equivalent	3 (25)	3 (25)	6 (25)
No qualifications		1 (8.33)	1 (4.17)
Annual household income before tax and deductions			
£10,001- £20,000		2 (16.67)	2 (8.33)
£ 20,001 - £30,000	2 (16.67)	2 (16.67)	4 (16.67)
£ 30,001 - £40,000	1 (8.33)	3 (25)	4 (16.67)
£ 40,001 - £50,000	2 (16.67)	1 (8.33)	3 (12.5)
£ 50,001 - £60,000	1 (8.33)		1 (4.17)
£ 60,001 - £70,000	1 (8.33)		1 (4.17)
£ 70,001 - £80,000	1 (8.33)	1 (8.33)	2 (8.33)
Prefer not to say	4 (33.34)	3 (25)	7 (29.17)
Years since Parkinson’s diagnosis *Mean (SD); Range*	6.91(5.39); 2–21		
Age at Parkinson’s diagnosis *Mean (SD); Range*	51.36 (7.51); 33–61		
Number of prescribed medications per day (medication types) *Mean (SD); Range*	3.9 (2.51); 1–9		
Number of tablets taken per day *Mean (SD); Range*	10.45 (6.73); 3–25		

* The* p*-values and χ2 documented here were obtained from independent sample t-tests and chi-squared test of independence which examined whether demographic characteristics significantly differ across participant groups

**Due to the number of respondents selecting non-white ethnicities to preserve anonymity here we categorised ethnicity as white and black and minority ethnicities.

*** Index of Multiple Deprivation (IMD) quintile. IMD is an index of neighbourhood deprivation generating one deprivation score for income, employment, education, health, crime, barriers to housing and services, and living environment. Quintile ‘1’ indicates the most deprived neighbourhoods, and quintile ‘5’ indicating the least deprived neighbourhoods [30]. See data codebook for further details regarding data transformation (https://osf.io/y8ve5/)

People with Parkinson’s were diagnosed between 2 and 21 years prior to their participation (*M* = 6.91(5.39)) and were aged between 33 and 61 at the time of diagnosis (*M* = 51.36(7.51)). Thus, although a substantial proportion, approximately 50%, of people with Parkinson’s were eligible for free prescriptions at the time of the focus group, all but one person with Parkinson’s had lived experience of having to pay for their prescriptions. All people with Parkinson’s were taking medication with people taking between 1 and 9 different types of medication a day. (See Table 1 for full sample characteristic breakdown).

We identified three over-arching themes with various sub-themes: (1) The financial toll of medication and its ripple effects; (2) Lack of inclusion and support; and (3) Difficulties of seeking support. (See Table 2 for a breakdown of themes and subthemes).

### The financial toll of medication and its ripple effects

The financial toll of prescription charges, in the context of Parkinson’s, appears to be multifaceted and extends beyond immediate financial burden. Many participants, both people with Parkinson’s and caregivers, expressed experiences of having to make lifestyle sacrifices to afford prescriptions, and not adhering to their medication regime. It is, however, important to note that these experiences were not unanimous. For instance, a few participants noted that the per-prescribed item charges did not place an additional burden.

#### Financial burden

Participants expressed difficulties in paying for prescriptions and managing other necessary expenses such as rent, bills and food expenses. There was a consensus that per-prescribed item charges increased feelings of anxiety and frustration, particularly for those on lower incomes.

*“Prescription charges for Parkinson’s disease hits hard on people*,* especially in low-income families. In my case*,* it sometimes gets hard to afford medication and the other stuff my family needs. I had to cut down on buying other things” (Caregiver (daughter)*,* Female*,* age 31–35*,* Participant 24)*.

*“It has quite an impact on me because having to get the medication at a high rate and also carry other expenses for the family like the food*,* school fees and other utility bills is quite difficult. Especially if you have to wait to get your salary at the end of the month and potentially some months before your salary is being paid. It’s been quite difficult for me to cope with” (Caregiver (daughter)*,* Female*,* age 36–40*,* Participant 23)*.

Although participants acknowledged that the prescription pre-payment certificate may serve to reduce financial burden, many participants expressed opinions that both the per-prescription and prescription pre-payment certificate fees are too costly and should be substantially reduced.

*“They should at least be reduced charges or totally exempt. If it’s a lifelong or terminal illness it should be dramatically reduced or totally exempt” (Person with Parkinson’s; Male*,* age 61–65*,* Participant 4)*.

*“I’ve heard of the prepayment plan and if you ask me it’s still quite a substantial amount*,* so I think the best way would be a reduction or maybe removal of the charges because medications are needed to live an independent and healthy life” (Caregiver (Son); Male*,* age 25–30*,* Participant 20)*.

While many people expressed experiences of financial burden, some participants noted that they have not financially struggled due to per-prescribed item charges. Such individuals accredited the lack of financial burden to either (a) being over the age of 60 and so being exempt from prescription charges, or (b) having financial security due to their occupation. Thus, suggesting an inequality in the impact of per-prescribed item charges.

*“So*,* the £10*,* £15*,* £20 a month*,* didn’t have a financial impact on me at all. But my family member*,* he’s 55 and he’s been diagnosed with Parkinson’s as well. I would think that if he starts going on multiple medications*,* even if he had to pay the £20 a month*,* that could have an impact on him cause he’s a single guy living in rented accommodation. So*,* for him*,* it might have more impact.” (Person with Parkinson’s; Male*,* age 61–65*,* Participant 9)*.

#### Lifestyle sacrifices

Many participants expressed that the immediate financial burden of per-prescribed item charges indirectly influenced their lifestyle choices. Some participants, mostly people with Parkinson’s, expressed experiences of having to forego activities they would normally engage in, to retain funds for per-prescribed item charges.

*“The way it [prescription charges] affected me made me change my kind of lifestyle. There were some things I wasn’t able to do anymore just to come up with money for my prescriptions.” (Person with Parkinson’s; Female*,* age 56–60*,* Participant 16)*.

*“I had to stop doing some of my hobbies*,* like my football*,* to save money back for the prescriptions.” (Person with Parkinson’s*,* Male*,* age 41–45*,* Participant 22)*.

One caregiver also expressed that the financial toll of per-prescribed item charges was so great that they had taken up additional employment to secure the funds required to meet the prescription charges for the person they care for.

*“It affected me in a way that I had to change my lifestyles reduce my bills and had to get a remote job to assist too.” (Caregiver(daughter); Female*,* Age 36–40*,* Participant 23)*.

#### Medication non-adherence

Participants also discussed that the financial burden of per-prescribed item charges has led them to deviate from their prescribed medication regimes. This sometimes included not collecting certain prescriptions or seeking out alternative, less costly, treatment options (e.g. herbal supplements).

*“The treatment is very difficult because of the high expenses. The person I care for actually had to skip medication because of the cost of medication which she was not able to afford.” (Caregiver(sister); Female*,* age 41–45*,* Participant 21)*.

*“We are pulling out so much money from our finances and it has had a lot of economic repercussions. Because of that*,* there are some medications that I don’t take… Oh*,* the repercussion is my family are hard.” (*Person with Parkinson’s, *Male*,* age 51–55*,* Participant 15)*.

*“The prescription of monthly medication impacted my life because at that point I could not actually afford the prescribed medications*,* I had to look for another alternative to help in calming the situation and handling it.” (*Person with Parkinson’s; *Female*,* age 56–60*,* Participant 16)*.

### Lack of inclusion and support

Many participants felt that current policies do not provide the support they need and that they are being let down by the system. They also highlighted inconsistencies in policy, such as which long-term health conditions and age groups qualify for exemptions, and the frustration these inconsistencies cause. Additionally, participants noted that awareness of available support is often by chance, with few organised efforts to assist people with Parkinson’s.

#### Failed by the system

Participants expressed sentiments that the omission of Parkinson’s in current medical exemption criteria leads to feelings of being let down by current policy and feeling hostage to the per-prescribed item charges.

*“It’s just like an extra tax. It’s like you get ill and pay some more tax…the disease is bad enough and now you are hostage to being charged for it.” (Person with Parkinson’s; Male*,* age 61–65*,* Participant 12)*.

Participants questioned whether these perceived inadequacies, of the per-prescribed item charge policy, in the context of Parkinson’s, are due to a general lack of understanding of Parkinson’s and the reliance people with Parkinson’s have on their medications. Importantly this lack of understanding was noted both at the level of medical professionals and policy makers.

*“How many GPs know how medications affect people with Parkinson’s? In my experience not many. So how can they inform on this?”(Caregiver(sister); Female*,* age 71–75*,* Participant 7)*.

*“Maybe there’s a lack of understanding about Parkinson’s. That’s the thing with government ministers and people who make the policy unless they are living with it [Parkinson’s] or living with somebody who’s got it [Parkinson’s]*,* they don’t really have a clue what impact it has on your daily life*,* the impact of it all.” (Caregiver(spouse); Male*,* age 66–70*,* Participant 5)*.

#### Inconsistencies in policy

Many participants expressed confusion due to the lack of consistency in the per-prescription charge exemption criteria. Participants questioned why Parkinson’s, like other long-term health conditions, is not included in this criterion and discussed the feelings of confusion and lack of support this omission generates.

*“It’s just I don’t see why Parkinson’s is not treated the same way as epilepsy. Medication is the lifeline for people with epilepsy*,* well it’s also my lifeline with Parkinson’s. Where’s the logic? It’s the largest neurological growing neurological disorder in the world. I know it’s behind Alzheimer’s*,* but it’s right up there. And you know what? Why has this never been on the exempt list and why isn’t it on the exempt list now?” (Person with Parkinson’s; Male*,* age 61–65)*.

Furthermore, participants expressed feelings that the medical exemption criteria appear to have been developed on an ad hock basis and are outdated. In light of these inconsistencies, several participants expressed feelings that the exemption criteria should be re-evaluated in its entirety.

*“I graduated in pharmacy in 1986 and the only big change in the exemptions in the entire lifetime of my career was that cancer was added. Everything else predated 1986…But that’s*,* you know*,* it’s just so outdated now. It needs completely re-evaluating” (*Person with Parkinson’s; *Female*,* age 56–60*,* Participant 10)*.

Several, participants also discussed the appropriateness of the age-based exemption criteria in the context of Parkinson’s. Participants discussed how they were required to pay per-prescribed item charges for numerous long-term medications for 15 to 20 years and experienced relief when hitting the age at which prescriptions became free.

*“If you are over 60 then you’re going to get a free prescription. In XX’s case*,* it was 16 years of paying for prescriptions before she was eligible. I am sure there are thousands of people out there right now in that situation.” (Caregiver(spouse); Male*,* age 66–70*,* Participant 5)*.

*“…from being in my early 40 s to being 60 I was certainly paying for my medication. And it was a joy coming off having to pay for it” (* Person with Parkinson’s; *Female*,* age 61–65*,* Participant 3)*.

#### Limited and by-chance awareness of support

Many participants, both people with Parkinson’s and caregivers, expressed that they only became aware of the prescription pre-payment certificate initiative through chance encounters. Indeed, some people reported becoming aware of pre-payment certificates by stumbling across leaflets or friends informing them.

*“I wasn’t aware of the prepayment card*,* whatever the official term is where you pay £20 a month. I found that out purely by accident from my best mate. He’s got motor neurone disease and he was on multiple medications and his missus said to me are you aware you can get this pre-payment certificate and I wasn’t so I looked at it….So first*,* I don’t think that’s advertised very well”(* Person with Parkinson’s; *Male*,* age 61–65*,* Participant 9)*.

Importantly, some participants first learned about the prescription pre-payment certificate during the focus group.

*“I am not on the prepayment plan because the information awareness is very low. I have not been on this plan but coming to hear about it today if I have my way I will try to be on it that will be so great” (* Person with Parkinson’s; *Female*,* age 56–60*,* Participant 19)*.

### Difficulties of seeking support

Although initiatives to reduce the financial burden of per-prescribed item charges are available, in our study, we discovered that the financial burden of prescription charges often extends beyond the individuals diagnosed with Parkinson’s to their families and caregivers. Some participants expressed that the cost of medication often needs to be covered out of their personal wages, putting a strain on both the individual and their support network. Although some participants noted receiving support from their communities and families, there was a shared sentiment of discomfort associated with seeking financial assistance for medication costs.

#### Reliance on family support

Several participants highlighted that they have had to rely on financial support provided by family members to finance their prescriptions. Some participants discussed the need for partial financial support (i.e. requiring financial support every now and then) whilst others indicated that they were entirely reliant upon financial support from family members.

*“In terms of prescriptions*,* we are only able to pay for them because of my brother’s support. So*,* he paid for them…I think there are about four or five medicines that are required and again they were so expensive for him to afford. So generally it’s not been easy but luckily one of my brothers has been supporting my mum in some way” (Caregiver(son); Male*,* age 31–35*,* Participant 14)*.

#### The burden of seeking support

There was a consensus, across both people with Parkinson’s and caregivers, that the act of seeking and struggling to find, financial support to cover per-prescription fees gave rise to feelings of discomfort and distress. As part of this, participants expressed feelings of unease in asking family for financial support, when they may be struggling to afford basic amenities due to the current cost of living crisis.

*“It makes me feel very uncomfortable due to the high cost of living. I feel uncomfortable asking for support with medication costs” (*Person with Parkinson’s; *Male*,* age 41–45*,* Participant 22)*.

## Discussion

Prior studies have independently shown the reliance on medication in people with Parkinson’s [9] and the impact of the cost of medications on individuals [13, 18–21]. However, this study was the first to directly investigate the impact of per-prescribed item charges on people with Parkinson’s and their caregivers, showing that the impact of per-prescribed item charges extends beyond immediate financial burden and also impacts people’s quality of life and medication adherence behaviours. There was an overarching disagreement with current policy and suggestions of per-prescription charge re-evaluation were expressed. Thus, this study’s findings considerably add to the existing literature.

People with Parkinson’s and caregivers expressed that they or the person they care for have previously deviated from their medical regime due to per-prescribed item costs. This is concerning as medication non-adherence in Parkinson’s can lead to poorer health outcomes including the more severe motor and non-motor symptoms, withdrawal symptoms and side effects including Parkinson’s hyperpyrexia syndrome which is potentially fatal [31, 32]. These poorer health outcomes may potentially directly impact the person’s quality of life [33] and may also have a direct impact on the health service providing care. Although not specifically related to Parkinson’s, recent estimates suggest that approximately 4.6% of total global health expenditures could be avoided with better medication adherence [34]. Moreover, through retrospectively analysing a USA patient claim database, Richy et al. [35] observed the mean costs associated with emergency health care and inpatient visits have been observed to be substantially higher in people who do not comply with their medication regime compared to those who comply. Thus, a lack of proper medication adherence in people with Parkinson’s, potentially facilitated by per-prescribed item charges, may indirectly increase medical demand and national medical expenditure. However, as the data collected here cannot directly speak to this hypothesis, further research directly exploring this link is required.

Both people with Parkinson’s and caregivers felt a lack of support within the current per-prescription policy, particularly due to the omission of Parkinson’s from the medical exemption criteria. Since its introduction in the 1960 s, the medical exemption criteria have only seen one substantial change, the addition of cancer [36]. The World Health Organisation estimated that the global incidence of Parkinson’s increased by 81% between 2000 and 2019 [37]. Indeed, Parkinson’s is now thought to be the fastest growing neurological condition in the world [2] affecting approximately 128,000 people in England alone [5]. Therefore, whilst Parkinson’s may not have been prevalent and pertinent to public health initiatives in the 1960 s when the medical exemption criteria was developed, the changes in the medical landscape render Parkinson’s increasingly important to public health initiatives in the 21 st century. As a result, the medical exemption criteria should be reinvestigated and updated. This study focused on people living with or caring for someone with Parkinson’s, therefore, the findings obtained relate only to Parkinson’s. However, it may indeed be the case that the sentiments expressed here are shared by people with different clinical conditions. Future research qualitatively exploring the experience of per-item prescription charges across a range of clinical conditions is required before further recommendations for changes to the policy are made.

It is indeed the case that the addition of medical conditions to the medical exemption criteria will reduce revenue to the NHS. However, a recent economic analysis suggested that the reduction in revenue, which would occur as a consequence of making prescriptions free for people with Parkinson’s, would be offset by savings to the NHS in England. Specifically, Hex et al. observed that savings of up to £0.8 m per year would be made if Parkinson’s alone was added to the medical exemption criteria [38].

Some participants questioned whether their feelings of a lack of support and the inequalities in the medical exemption criteria in part arose due to people in a position of power lacking a clear understanding of what Parkinson’s is and how it affects people daily. A recent meta-analysis concluded that whilst global awareness of Parkinson’s was adequate, an understanding of the wealth of symptoms, beyond the tremor associated with Parkinson’s and its treatment is lacking [39]. This is corroborated by findings from this study. Disclosure of the information drawn upon during the development of the medical exemption criteria would allow for a greater understanding of the government’s justification of a lack of inclusion of many long-term health conditions and thus may reduce such feelings in people with Parkinson’s and caregivers.

Prior research has shown that public awareness of the prescription pre-payment certificate is low to moderate [13]. Moreover, people who are aware of and use the prescription pre-payment certificate often do not become aware of it for a substantial period, typically between 6 months to 1 year following long-term health condition diagnosis [14]. Supporting this previous evidence, participants in this study expressed that awareness of support is low, indeed some participants were unaware of the prescription pre-payment certificate initiative up until the point of their participation. Importantly, awareness, in this specific sample, appears to be driven by chance encounters including but not limited to friends having prior knowledge of the prescription pre-payment certificate and participants seeing a flyer in a medical setting. Subsequently, it may be beneficial to increase visual and verbal advertising presence in locations frequently attended by people who would benefit from the pre-payment certificate including doctor’s surgeries, hospitals, outpatient services, pharmacies and potentially high street locations, for example, supermarkets and post offices.

These findings may have several important implications for per-prescribed item charge policy and clinical practice. Collectively people with Parkinson’s and their caregivers indicated that they believe current per-prescription charges to be too costly, and lifestyle sacrifices, and a lack of medication adherence were discussed as consequences of such fees. As such, it would be beneficial for the current per-prescribed item charge policy to be thoroughly reviewed and updated. Participants, both people with Parkinson’s and caregivers, felt a lack of support by current policy due to the omission of Parkinson’s from the medical exemption criteria. Presently, there is a lack of consistency in the clinical conditions that are included in the medical exemption criteria, nor is there a clear justification for the exclusion of some conditions in this criterion. Re-evaluating the medical exemption criteria and justifying the clinical conditions included would provide clarity and potentially reduce feelings of a lack of support.

This study has strengths. Some of these include its ongoing involvement of people with Parkinson’s and their caregivers as public advisers, the diversity of the researchers and the diversity in demographics of the participants in terms of employment status, occupation and income. However, it is not without its limitations. First, a greater proportion of people from white ethnic backgrounds and highly educated people participated in this study. Socioeconomic and demographic factors, including both ethnicity and educational attainment, are known to influence healthcare initiative awareness [40] and utilisation [41]. Therefore, these findings may not be theoretically transferable to the groups that populations that were not as well represented in the sample. Moreover, as direct access to participants’ medical records was not obtained for the purpose of this study, participant eligibility was based on self-disclosed medical information. Due to differences in the clinical management of alternative types of Parkinson’s and Parkinsonism’s, compared to idiopathic Parkinson’s, only people with idiopathic Parkinson’s were eligible to participate. It is possible, however, that some individuals are not aware of the precise type of Parkinson’s they have, moreover, it is not uncommon for people living with Parkinson’s and Parkinsonism’s to receive an alternative diagnosis as the trajectory of their condition alters [42]. As such, we cannot firmly conclude people living with alternative types of Parkinson’s and Parkinsonism’s.

Second, considerable active effort was made to recruit people with Parkinson’s and caregivers of people with Parkinson’s who are currently paying for their prescriptions. However, in practice, many of the participants recruited were no longer paying for their prescriptions and therefore reflected on past experiences. In the UK, consumer prices have drastically risen over the past three years, leading to a cost of living crisis and a significant decline in disposable income. In line with this, the cost of per-prescribed item charges has increased 8.8% between 2020 and 2024. Thus, it may be that the past experiences of people living with or caring for people with Parkinson’s who had to pay for their prescriptions several years ago do not reflect the experiences of people living with or caring for people with Parkinson’s who have to pay for their prescriptions presently, in 2024. Nonetheless, as the impact of pre-prescribed items on people living with and caring for people with Parkinson’s has not been explored before we find the perspectives of this population to be of upmost importance. Future research should aim to recruit a sample of participants who all currently pay for their prescription charges to ascertain whether experiences have changed over time.

Finally, this study recruited only people with Parkinson’s and caregivers of people with Parkinson’s. Parkinson’s is a complex and unique clinical condition in which pharmacological intervention remains the gold standard treatment and hence is relied upon. Therefore, it could be that the findings obtained here may not reflect the experience of people living with alternative long-term health conditions in which reliance on pharmacological intervention is less pronounced. Therefore, future studies that employ comparable methodologies with alternative clinical populations are required to ascertain whether the findings obtained here are unique to people with Parkinson’s or are representative of the wider long-term health condition population.

## Conclusion

Findings indicate that per-prescribed item charges impact both people with Parkinson’s and caregivers in a multifaceted manner. Impact extends beyond the need for immediate financial sacrifices and may impact people’s quality of life, through foregoing activities and taking up additional employment to reserve funds for prescriptions, cause distress, through the act of seeking support, and reduce medication adherence. These findings have important implications for prescription charge policy. Specifically, given that per-prescribed item charges give rise to substantial personal, and potentially societal, impacts it would be beneficial for current prescription charge policy, including both the level of charge and medical exemption criteria, to be reviewed.

## Supplementary Information

Supplementary Material 1.

## Data Availability

Data from this study are available from the corresponding author (MR; m.readman@liverpool.ac.uk) on request. The raw data have not been uploaded to the open science framework due to ethical limitations.
