# Supplementary material for: Understanding the experience of prescription charges in people living with parkinson’s disease: a focus group study
Source: BMC Public Health. 2025 Aug 22;25:2879. doi: 10.1186/s12889-025-24121-0 (PMC12372227; doi:10.1186/s12889-025-24121-0)
Supplement: Supplementary file 1 — Supplementary Material 1. [file 12889_2025_24121_MOESM1_ESM.docx]

**Supplementary Materials**

**Contents**

1. Justification of deviations from OSF Pre-registration 2
2. Focus Group Schedule 3
3. Consolidated criteria for reporting qualitative studies (COREQ): 32-item checklist

**Justification of deviations from OSF Pre-registration**

This study deviated from the protocol only in the proportion of total number of participants recruited and the transformations applied to postcode and occupation data. Specifically, we aimed to recruit a purposive sample of 25 people (~ 15 people living with Parkinson’s and 10 family care partners (caregivers) across the 5 focus groups. However, at the point of data saturation we had recruited 24 people (12 people with Parkinson’s and 12 caregivers).

Moreover, we planned to not transform the postcode and occupation data and present standard summary statistics. However, to aid interpretability, we have transformed the postcode data to Indices of Multiple Deprivation and recoded the occupation data to conform to the Standard Occupational Classification: SOC2020 criteria [43, 44]. Moreover, to better visualise the geographical spread of the participants, and maintain participant anonymity, postcodes were transformed to local authority data (i.e. the local authority in which the participant resides). Further information regarding variable transformations can be found in the OSF documentation (<https://osf.io/y8ve5/>).

**Version Number:** 1.1

**Date of this version:** 15/06/2023

**Focus group Protocol**

**Focus group #:______________**

**Pre-interview checklist for interviewer:**

**☐** Participant information sheet reviewed by all attendees

**☐** Consent process completed by all attendees

**Introduction**

Thanks for agreeing to take part in this focus group for our study today.

[*Moderator introduce themselves. Include job title, research interests, who they work for. I’d also like to introduce (NAMES OF CO-MODERATOR, OTHERS]*

As mentioned in the information sheet and consent form this focus group will be audio recorded. So, if it is okay with you all I will now begin recording.

[SWITCH ON AUDIO RECORDER]

Ground Rules for Group:

Let me give you a few quick ground rules for the focus group. First, everyone’s opinion is valued and it’s OK to disagree with each other or with me and the other co-moderators. We are very interested in hearing about all points of view. It’s OK to talk to each other and not just to me. It’s OK to get up for more coffee or to go to the restroom and since our time is limited I may need to ask you to stop and change topics from time to time

Introduction of Participants:

Now I’d like to go around the table so each person can give me their first name only and tell me in a few words something they did this week that was important or fun. Again, no last names or other information that would identify you and keep it short, please.

Introduction of Subject of Group:

In this study we aim to understand more about the experiences of prescription charges for people living with Parkinson’s disease and their families. Specifically, we aim to understand more about how prescription charges may impact the general wellbeing and mental health of people living with Parkinson’s and their families. Some of the specific things we would like you to think about are your medication taking behaviours (i.e. how often you take medications and how many medications) and how medication consumption and prescription charges may have influenced your life in any way. We are interested in your experiences regarding prescription medication and their charges, and those you have seen perhaps in friends. In particular, if you were diagnosed with Parkinson’s before the age of 60, we would like you to think about your experiences both now and before 60. If you were not diagnosed before 60 we would like you to think about the experience of people you may know who were diagnosed before the age of 60 as well as your own experiences. We would also like you to consider what you would like to see regarding prescription medication charges for people living with Parkinson’s and other related long-term conditions. How would you like to see policy be shaped?

| Involvement exercise  *Discussion begins (below), make sure to give people time to think before answering the questions and don’t move too quickly. Use the probes to make sure that all issues are addressed, but allow for flexibility in the direction that the participant takes the conversation* | | |
| --- | --- | --- |
| **Check** | **#** | **Probe** |
| ☐ | 1 | Firstly, we would like to think about and discuss your experiences of taking prescription medication during the course of your Parkinson’s. Did you commence taking medication straight away? Were you faced with any charges?  Additional prompts:   - How do you feel about the prescription charges? - How have these charges changed over time? - Have these prescription charges impacted your life in any way? - Have these prescription charges impacted on other needs and if so how have you coped with this? |
| ☐ | 2 | Now we would like you to think about how these charges make you feel and have they may have impacted you in any way?  Additional prompts   - How do you feel about the prescription charges? - Have these prescription charges impacted your life in any way? - Have these prescription charges impacted on other needs and if so how have you coped with this? |
| ☐ | 3 | Now we have established everyone’s experiences, we would like you to discuss what the issue is here. What do you think are the issues surrounding prescription medication charges and the process of obtaining medications in the specific context of Parkinson’s ?  Additional prompts:   - What do you think the key challenges are? - What do you think the missed opportunities are? - Do you have any pet peeves regarding this matter? |
| ☐ | 4 | Now that we have collectively identified the problem in this space, I would like you to turn your heads to the solution. Specifically what you would like to see improved?  Additional prompts:   - If money was no object, what would you like to see improved? |
| Thank you for your time. I’m going to switch off the recorder now. [SWITCH OFF AUDIO RECORDER] | | |
| **Data collection diary** | | |
| *Add anonymous comments only, to aid interpretation of transcripts* | | |

**Consolidated criteria for reporting qualitative studies (COREQ): 32-item checklist**

| **No. Item** | **Guide questions/description** | **Reported on Page #** |
| --- | --- | --- |
| **Domain 1: Research team and reﬂexivity** |  |  |
| *Personal Characteristics* |  |  |
| 1. Inter viewer/facilitator | Which author/s conducted the inter view or focus group? | Page 5 |
| 2. Credentials | What were the researcher’s credentials? E.g. PhD, MD | Page 1 |
| 3. Occupation | What was their occupation at the time of the study? | Page 6 |
| 4. Gender | Was the researcher male or female? | Page 5 |
| 5. Experience and training | What experience or training did the researcher have? | Page 5 |
| *Relationship with participants* |  |  |
| 6. Relationship established | Was a relationship established prior to study commencement? | Page 6  . |
| 7. Participant knowledge of the interviewer | What did the participants know about the researcher? e.g. personal goals, reasons for doing the research | Page 6 |
| 8. Interviewer characteristics | What characteristics were reported about the inter viewer/facilitator? e.g. Bias, assumptions, reasons and interests in the research topic | Page 6 |

| **Domain 2: study design** |  |  |
| --- | --- | --- |
| *Theoretical framework* |  |  |
| 9. Methodological orientation and Theory | What methodological orientation was stated to underpin the study? e.g. grounded theory, discourse analysis, ethnography, phenomenology, content analysis | Page 5 |
| *Participant selection* |  |  |
| 10. Sampling | How were participants selected? e.g. purposive, convenience, consecutive, snowball | Page 4 |
| 11. Method of approach | How were participants approached? e.g. face-to-face, telephone, mail, email | Page 4 |
| 12. Sample size | How many participants were in the study? | Page 6 |
| 13. Non-participation | How many people refused to participate or dropped out? Reasons? | Page 6 |
| *Setting* |  |  |
| 14. Setting of data collection | Where was the data collected? e.g. home, clinic, workplace | Page 5  . |
| 15. Presence of non-participants | Was anyone else present besides the participants and researchers? | Page 5 |
| 16. Description of sample | What are the important characteristics of the sample? e.g. demographic data, date | Page 6 and Table 2 |
| *Data collection* |  |  |
| 17. Interview guide | Were questions, prompts, guides provided by the authors? Was it pilot tested? | Page 4-5 and supplementary material |
| 18. Repeat interviews | Were repeat inter views carried out? If yes, how many? | No, page 5 |
| 19. Audio/visual recording | Did the research use audio or visual recording to collect the data? | Page 5 |
| 20. Field notes | Were ﬁeld notes made during and/or after the inter view or focus group? | Page 5 |
| 21. Duration | What was the duration of the inter views or focus group? | Page 5 |
| 22. Data saturation | Was data saturation discussed? | Page 6-7 |
| 23. Transcripts returned | Were transcripts returned to participants for comment and/or correction? | Page 5 |
| **Domain 3: analysis and ﬁndings** |  |  |
| *Data analysis* |  |  |
| 24. Number of data coders | How many data coders coded the data? | Page 5 |
| 25. Description of the coding tree | Did authors provide a description of the coding tree? | Page 5 and Table 1 |
| 26. Derivation of themes | Were themes identiﬁed in advance or derived from the data? | Page 5 |
| 27. Software | What software, if applicable, was used to manage the data? | Page 5 |
| 28. Participant checking | Did participants provide feedback on the ﬁndings? | Page 5-6 |
| *Reporting* |  |  |
| 29. Quotations presented | Were participant quotations presented to illustrate the themes/ﬁndings? Was each quotation identiﬁed? e.g. participant number | Page 7 to 11 |
| 30. Data and ﬁndings consistent | Was there consistency between the data presented and the ﬁndings? | Yes, there was.  Page 7 to 11 |
| 31. Clarity of major themes | Were major themes clearly presented in the ﬁndings? | Yes. they were.  From page 7 to 11 |
| 32. Clarity of minor themes | Is there a description of diverse cases or discussion of minor themes? | Discussion of major and minor themes  From page 7 to 11 |
